# Association between 3801T>C Polymorphism of CYP1A1 and Idiopathic Male Infertility Risk: A Systematic Review and Meta-Analysis

**DOI:** 10.1371/journal.pone.0086649

**Published:** 2014-01-21

**Authors:** Haiqing Luo, Hongjiao Li, Na Yao, Liren Hu, Taiping He

**Affiliations:** 1 Center of Oncology, The Affiliated Hospital of Guangdong Medical College, Zhanjiang, China; 2 Department of Biochemistry and Molecular Biology, Guangdong Medical College, Zhanjiang, China; 3 Department of Epidemiology and Health Statistics, School of Public Health, Guangdong Medical College, Zhanjiang, China; 4 School of Public Health, Guangdong Medical College, Zhanjiang, China; National Cancer Center, Japan

## Abstract

**Background:**

Epidemiological studies have evaluated the association between 3801T>C polymorphism of CYP1A1 gene and the risk for idiopathic male infertility, but the results are inconclusive. We aimed to derive a more precise estimation of the relationship by conducting a meta-analysis of case-control studies.

**Methods:**

This study conformed to Preferred Reporting Items for Systematic Reviews and Meta-Analyses guidelines. PubMed, Embase and CNKI databases were searched through November 2013 to identify relevant studies. Pooled odds ratios with 95% confidence intervals were used to assess the strength of the association between CYP1A1 3801T>C polymorphism and idiopathic male infertility risk. *Q*-test was performed to evaluate between-study heterogeneity and publication bias was appraised using funnel plots. Sensitivity analyses were conducted to evaluate the robustness of meta-analysis findings.

**Results:**

Six studies involving 1,060 cases and 1,225 controls were included in this meta-analysis. Overall, significant associations between 3801T>C polymorphism and idiopathic male infertility risk were observed in allelic comparison (OR = 1.36, 95% CI: 1.01–1.83), homozygous model (OR = 2.18, 95% CI: 1.15–4.12), and recessive model (OR = 1.86, 95% CI: 1.09–3.20), with robust findings according to sensitivity analyses. However, subgroup analyses did not further identify the susceptibility to idiopathic male infertility in all comparisons. Funnel plot inspections did not reveal evidence of publication bias.

**Conclusions:**

The current meta-analysis provides evidence of a significant association between CYP1A1 3801T>C polymorphism and idiopathic male infertility risk. Considering the limitation inherited from the eligible studies, further confirmation in large-scale and well-designed studies is needed.

## Introduction

Idiopathic male infertility (IMI) is a global health dilemma affecting approximately 10–15% of the male adults and up to one in six couples, which accounts for 40–50% male infertility problems [1]–[3]. The causes of IMI are the result of complex interaction of multi-factorial and environmental, behavioral and genetic factors [4]. Despite progresses in diagnosis of IMI, mainly in the field of genetics, etiology and pathogenesis are still unknown. Epidemiological data indicated that damage of xenobiotic to the genetic material might contribute to spermatogenetic failure for about 30% of infertility in males [5]. A number of genetic studies have been done to investigate the contribution of genes encoding to IMI, and some of these studies have revealed the direct relationship between the genotypes and the disease susceptibility [6], [7]. Much attention in the genetic studies has been paid to the cytochrome P450 1A1 (CYP1A1) that plays a curial role in phase I metabolism of polycyclic aromatic hydrocarbons to their ultimate biologically active intermediates that have potential reproductive toxicity in men [5], [8]. The CYP1A1 gene has been shown to be polymorphic, and several polymorphisms have been identified [9]. Among these polymorphisms, the most commonly studied is the 3801T>C polymorphism (also referred to as 2A, m1, or rs4646903), which is characterized by the T to C mutation at nucleotide 3801 in the 3′ flanking region of the CYP1A1 gene. The 3801T>C polymorphism can alter the level of gene expression or messenger RNA stability, resulting in a highly inducible activity of the enzyme [10], [11]. To date, several studies have investigated the relationship between the CYP1A1 3801T>C polymorphism and IMI risk. However, current results have been inconsistent. Moreover, no meta-analysis data on the correlation of the polymorphism with susceptibility to IMI is currently available. Therefore, to derive a more precise overall effect estimate, the present study aimed to evaluated the association between the CYP1A1 3801T>C polymorphism and susceptibility to IMI by performing a systematic review and meta-analysis of the literature.

## Materials and Methods

### Literature search

This systematic review and meta-analysis followed Preferred Reporting Items for Systematic Reviews and Meta-Analyses (PRISMA) guidelines. The included studies were retrieved from the electronic databases including PubMed, Embase and China National Knowledge Infrastructure (CNKI) databases. We used the combination of the search terms: (“CYP1A1” or “cytochrome P450 1A1” or “rs4646903”), (“idiopathic male infertility” or “male infertility” or “oligozoospermia” or “azoospermia” or “teratospermia”) and (“polymorphism” or “allele” or “variant” or “mutation” or “gene” or “genotype”). No restrictions were imposed on the search in terms of language. The literature search was updated on 20 November 2013.

### Study selection

The studies were selected according to the following inclusion criteria: (1) used a case-control design, (2) gave information on the distribution of CYP1A1 3801T>C genotypes in both cases and corresponding controls, (3) contained an evaluation of the 3801T>C polymorphism and IMI risk, and (4) consisted with Hardy-Weinberg equilibrium (HWE) in control groups. We excluded those studies that did not provide adequate information on selection criteria and in which allele frequencies in controls exhibited significant deviation from the HWE (*P*<0.05). Conference abstracts, case reports, editorials, review articles, and letters were also excluded.

### Data extraction

Information was extracted from all eligible publications independently by two of the authors according to the above-listed inclusion criteria. An agreement was reached through a discussion between the two reviewers for cases with conflicting information. The following characteristics were collected from each study: the first author's name, publication year, country, population ethnicity, and genotype frequency for cases and controls. Populations from the included studies were stratified into Caucasian and Asian. Notably, Indians are mainly of Indo-European and Dravidian ancestries, which are quite different from East Asians [12]. From the point of view of history and geography, Iran lies on the route of major ancient movements of the Caucasian people towards the Mediterranean basin, Iranian should be descendants of European [13]. Moreover, Indian and Iranian were usually categorized into Caucasian in previous publications [14]. Thus, the two populations in the meta-analysis were also stratified into Caucasian.

### Quality score evaluation

Quality assessment of the case-control studies in this meta-analysis was performed using the Newcastle Ottawa scale (NOS) recommended by the Cochrane Non-randomized Studies Methods Working Group [15], [16]. The NOS contains eight items that are categorized three categories: selection (four items, one star each), comparability (one item, up to two stars), and exposure (three items, one star each). A “star” presents a “high-quality” choice of individual study. Two independent reviewers discussed their evaluation, and any disagreements were resolved through discussion and consultation. Given the variability in quality of case-control studies found in our initial literature search, we considered studies as high quality if they met a score of six or more of the NOS criteria [17].

### Statistical analysis

STATA version 11.0 (STATA Corporation, College Station, Texas) was used for all statistical analyses. All statistical tests were two sided. The combined odds ratio (OR), along with its corresponding 95% confidence interval (CI), was used to calculate and assess the strength of the association between polymorphisms of CYP1A1 and IMI risk. The allele model, co-dominant model (heterozygous carriers vs. “wild type” and homozygous carriers vs. wild type”), dominant model (heterozygous and homozygous carriers grouped together vs. “wild type”), and recessive model (homozygous carriers vs. “wild type” and heterozygous carriers grouped together) were estimated [18].

Heterogeneity assumption was examined using the *Q*-test [19]. A random-effects model (DerSimonian-Laird method) or fixed-effects model (Mantel-Haenszel method) was used to calculate the pooled effect estimates in the presence (*P*< = 0.10) or absence (*P*>0.10) of heterogeneity, respectively [20]. To explore the reasons of heterogeneity, subgroup analyses were performed by grouping studies that showed similar characteristics, such as ethnicity, sample size, and quality assessment score. Sensitivity analysis was also performed by omitting each individual study to reflect the influence of the individual dataset on the pooled OR using the “metaninf” STATA command. The appropriate Chi-square goodness-of-fit test [21] was performed using the “genhwcci” STATA command to assess the deviation from HWE only in control groups. Statistical significance for the interpretation of the chi-squared test was defined as *P*<0.05.

Publication bias was evaluated through the Begg's and the Egger's Asymmetry tests [22] and through visual inspection of funnel plots, in which the standard error was plotted against the log (OR) to form a simple scatterplot. Statistical significance for the interpretation of the Egger's test was defined as *P*<0.10.

## Results

### Eligible studies

**Figure 1:**
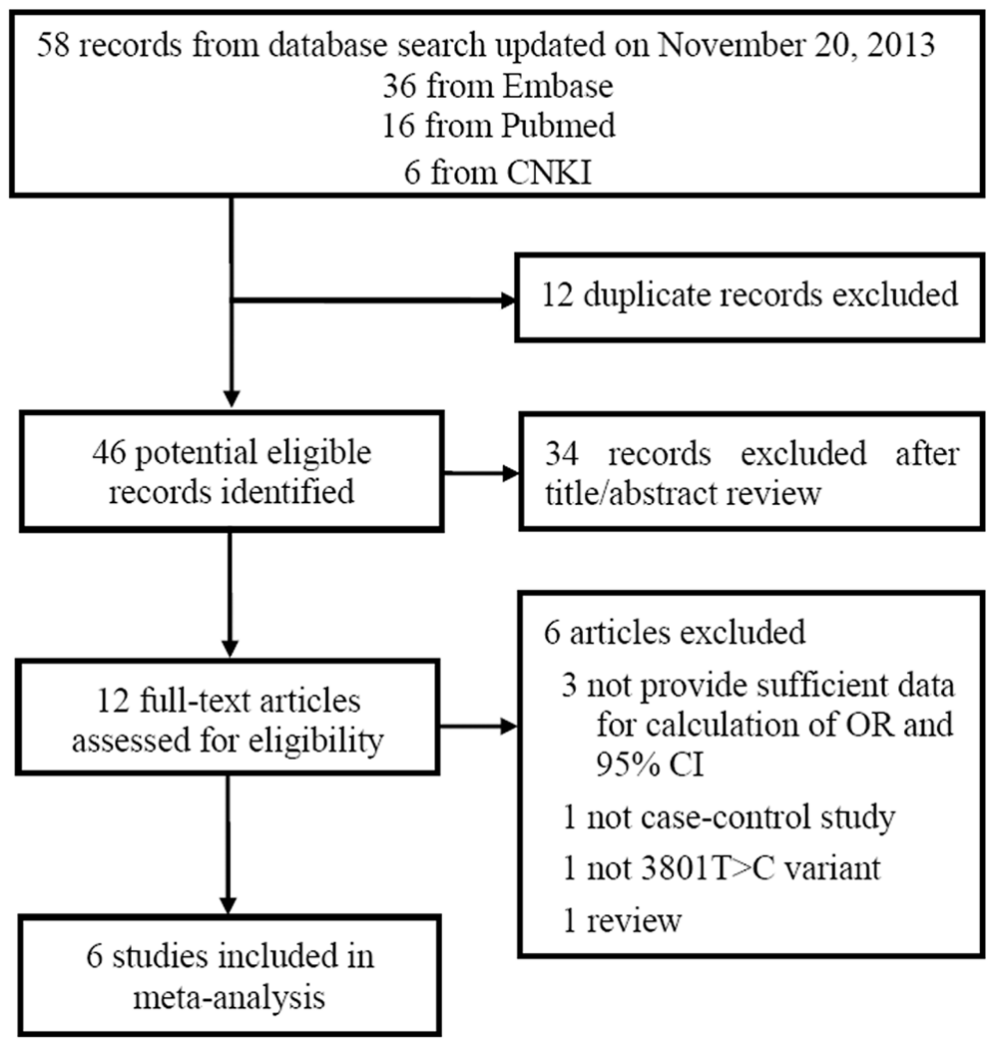
Flow diagram of study selection and specific reasons for exclusion in the meta-analysis.

Figure 1 illustrates the trial flow diagram for study inclusion. A total of 58 citations were identified during the initial search. Then 12 duplicate records were excluded. Of the 46 potential eligible records, 34 were excluded because of obvious irrelevance by reading their titles and abstracts (reasons for exclusion described in Table S1). After detailed evaluation, the following were excluded: one review [5], one not in a case-control design [23], one not of reporting 3801T>C polymorphism [24] and three articles that did not provide sufficient data for calculation of OR and 95% CI [8], [25], [26]. Finally, six articles [27]–[32] met the inclusion criteria involving 1,060 cases and 1,225 controls. In the articles eligible for the meta-analysis, three [28], [31], [32] were conducted in Caucasian populations, and three [27], [29], [30] involved Asian populations. For the determination of the genetic polymorphism, polymerase chain reaction-restriction fragment length polymorphism was used in all the studies. Semen specimens of cases were diagnosed primarily according to WHO guidelines. Control subjects were from hospital-based populations in all of the included studies. Five studies [27]–[31] stated that the controls were age matched, and one [32] was matched by smoking status. Noticeably, Lu et al. [27] didn't report age of cases and controls, but we read the author's academic dissertation published in Chinese and found that the population was age-matched. Salehi et al. [31] was age-matched too, although without detailed information about age. Examining the genotype frequencies in controls, no significant deviation from HWE was detected in all studies (all *P*>0.05). The studies were published between 2008 and 2013. A wide variation of the 3801C allele frequencies across different populations was observed, varying from 0.12 in Russians to 0.38 in Chinese. Four studies were of high quality with score greater than 6 (details of evaluation score for literature shown in Table S2). The characteristics of the case–control studies included for the polymorphism are summarized in Table 1 and S3.

**Table 1:** CAYP1A1 3801T >C genotype distribution and allele frequencies in cases and controls.

Study	Year	Country	Ethnicity	Control source	Genotyping method	Cases/controls	Genotype data (cases)					Genotype data (controls)					MAF	HWE (P)^a^	Quality score
							T	C	TT	TC	CC	T	C	TT	TC	CC			
Lu et al [27]	2008	China	Asian	HB	PCR-RFLP	192/226	234	150	69	96	27	294	158	95	104	27	0.35	0.86	6
Vani et al [28]	2009	India	Caucasian	HB	PCR-RFLP	206/230	296	116	108	80	18	372	88	146	80	4	0.19	0.06	8
Chen et al [29]	2010	China	Asian	HB	PCR-RFLP	105/140	117	93	35	47	23	221	59	88	45	7	0.21	0.69	5
Peng et al [30]	2012	China	Asian	HB	PCR-RFLP	204/202	237	171	72	93	39	250	154	78	94	30	0.38	0.85	5
Salehi et al [31]	2012	Iran	Caucasian	HB	PCR-RFLP	150/200	188	112	58	72	20	261	139	85	91	24	0.35	0.96	6
Yarosh et al [32]	2013	Russia	Caucasian	HB	PCR-RFLP	203/227	365	41	165	35	3	400	54	176	48	3	0.12	0.89	8

*HB* hospital based, *PCR-RFLP* polymerase chain reaction-restriction fragment length polymorphism, *MAF* minor allele frequency, *HWE* Hardy–Weinberg equilibrium,

a P value for HWE in control group.

### Quantitative synthesis

**Figure 2:**
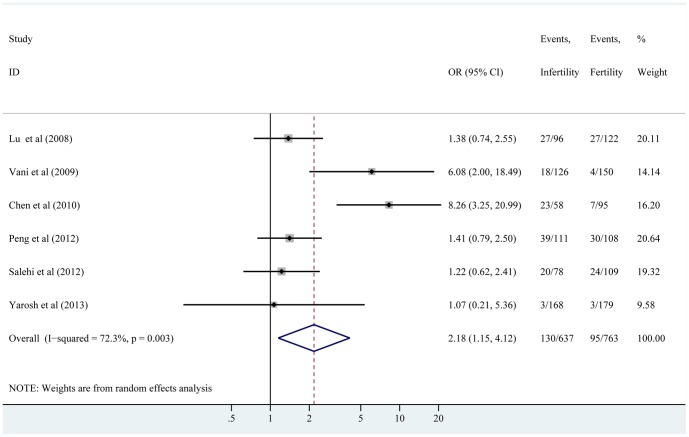
Meta-analysis of the association between the 3801T>C polymorphism of CYP1A1 and risk for idiopathic male infertility under homozygous model. The contribution of each study to the meta-analysis (its weight) is represented by the area of a box, the center of which represents the size of the OR estimated from that study. The 95% CI for the OR (extending lines) from each study is also shown. The overall OR is shown in the middle of a diamond, the left and right extremes of which represent the corresponding CI.

The pooled OR with its 95% CI, are presented in detail in Table 2. Overall, significant associations were demonstrated in allele model (C vs. T, OR = 1.36, 95% CI: 1.01–1.83), homozygous model (CC vs. TT, OR = 2.18, 95% CI: 1.15–4.12), and recessive model (CC vs. TT+TC, OR = 1.86, 95% CI: 1.09–3.20). However, null significant association was observed in heterozygous model (TC vs. TT, OR = 1.25, 95% CI: 0.95–1.65) and dominant model (CC+TC vs. TT, OR = 1.38, 95% CI: 0.99–1.92). A further analysis was performed on data stratified by ethnicity to determine possible factors that might have influenced the results. Table 2 shows that any association was not found in IMI patients with CYP1A1 3801T>C polymorphism among all comparisons. Moreover, similar results in the further subgroup analysis by quality score and sample size were observed on any genetic model because the associations did not reach a significant level. The forest plots for the overall association between the 3801T>C polymorphism of CYP1A1 and IMI risk are shown in Figure 2 and S1.

**Table 2:** Results of meta-analysis for 3801T>C polymorphism of CYP1A1 and idiopathic male infertility risk.

Study group	No. of study	sample size (cases/controls)	Allele model (C vs. T)		Homozygous model (CC vs. TT)		Heterozygous model (TC vs. TT)		Dominant model (CC+TC vs. TT)		Recessive model (CC vs. TC+TT)	
			OR (95% CI)	*P*_h_	OR (95% CI)	*P*_h_	OR (95% CI)	*P*_h_	OR (95% CI)	*P*_h_	OR (95% CI)	*P*_h_
All	6	1060/1225	**1.36(1.01,1.83)**	<0.001	**2.18(1.15,4.12)**	0.003	1.25(0.95,1.65)	0.051	1.38(0.99,1.92)	0.003	**1.86(1.09,3.20)**	0.013
Asian	3	501/568	1.58(0.93,2.67)	<0.001	2.35(0.90,6.16)	0.003	1.48(0.91,2.40)	0.040	1.67(0.92,3.05)	0.004	1.90(0.89,4.05)	0.015
Caucasian	3	559/657	1.18(0.81,1.72)	0.031	2.02(0.66,6.20)	0.042	1.13(0.89,1.43) ^F^	0.353	1.16(0.79,1.71)	0.086	1.89(0.64,5.58)	0.046
High quality	4	751/883	1.19(0.93,1.53)	0.073	1.75(0.89,3.43)	0.028	1.15(0.92,1.42) ^F^	0.339	1.22(0.99,1.50) ^F^	0.170	1.58(0.83,3.00)	0.084
Low quality	2	309/342	1.85(0.74,4.61)	<0.001	3.28(0.58,18.56)	0.002	1.64(0.68,3.96)	0.013	1.95(0.68,5.59)	0.002	2.56(0.67,9.78)	0.009
Sample size >400	4	805/885	1.21(0.95,1.53)	0.081	1.79(0.97,3.32)	0.100	1.13(0.91,1.39) ^F^	0.330	1.20(0.92,1.56) ^F^	0.167	1.55(0.98,2.19) ^F^	0.107
Sample size <400	2	255/340	1.81(0.69,4.73)	<0.001	3.09(0.47,20.14)	<0.001	1.71(0.77,3.82)	0.027	1.86(0.59,55.89)	0.001	2.37(0.52,10.89)	0.005

*P_h_ P* value for heterogeneity based on Q test. All pooled ORs were derived from random-effects model except for cells marked with (fixed ^F^).

### Evaluation of heterogeneity

We analyzed the heterogeneity of the studies selected according to the *P*-value for heterogeneity. Table 2 demonstrates that a significant heterogeneity was found in all genetic models (*P*_heterogeneity_<0.10). Therefore, a random-effects model was used for all the comparisons to calculate the overall OR estimates. After assessing the source of heterogeneity for all genetic models compared by subgroup analyses based on ethnicity, quality score, or sample size, the heterogeneity was partly decreased or removed.

### Sensitivity analysis

**Figure 3:**
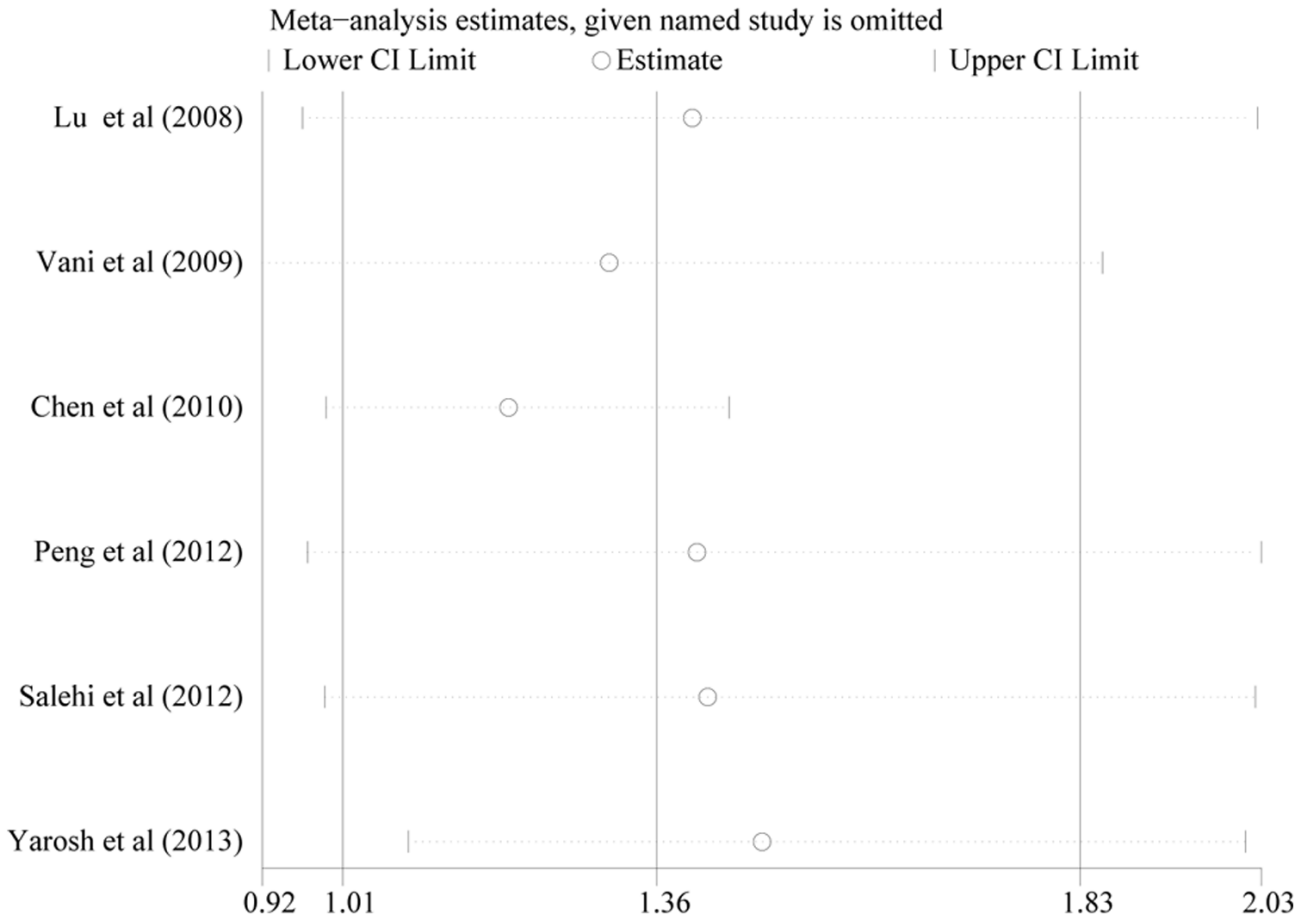
Effect of individual studies on the pooled OR under allele comparison for the 3801T>C polymorphism of CYP1A1 in idiopathic male infertility. The vertical axis at 1.36 indicates the overall OR, and the two vertical axes at 1.01 and 1.83 indicate the 95% CI. Every hollow round indicates the pooled OR when the left study was omitted in a meta-analysis with a random model. The two ends of every broken line represent the respective 95% CI.

Separate meta-analyses were conducted to investigate the influence of each individual study on the overall meta-analytic estimate. Figure 3 demonstrates that no point estimate of the omitted individual study lay outside the 95% CI of the combined analysis on the allele model. Similarly, no significant influence was observed when an analysis was conducted on the other models (Figures were not shown). These analyses suggest that no individual study affected the results in the meta-analysis.

### Publication bias

**Figure 4:**
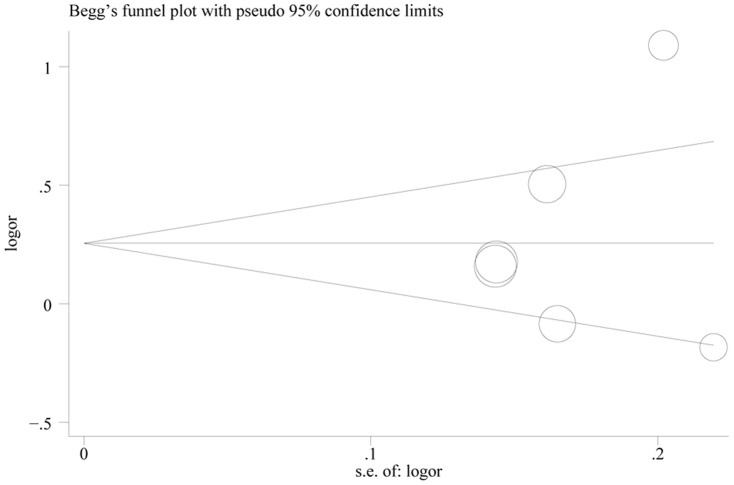
Funnel plot for the 3801T>C polymorphism of CYP1A1 under allele comparison in idiopathic male infertility. The vertical axis represents log (OR), and the horizontal axis refers to the standard error of log (OR). The horizontal line indicates the pooled OR, and the sloping lines indicate the expected 95% CI for a given standard error. The area of each circle represents the contribution of the study to the pooled OR.

No publication bias on the overall OR analysis was detected at any comparison (Table 3, *P*>0.10). The shapes of the funnel plots appeared to be roughly symmetrical in all genetic models (Figure 4 and S2). In addition, neither the Begg's test nor the Egger's test provided any obvious evidence of publication bias in subgroup analysis (data was not shown).

**Table 3:** Results of Egger's test and Begg's test.

Comparison	Egger's test				Begg's test	
	*t*	*P*	95% CI		*Z*	*P*
Allele model	0.42	0.697	−16.480	22.339	0.00	0.999
Homozygous model	0.72	0.514	−6.184	10.475	0.38	0.707
Heterozygous model	0.87	0.432	−10.697	20.504	0.38	0.707
Dominant model	0.82	0.461	−14.594	26.723	0.38	0.707
Recessive model	1.26	0.277	−2.986	7.929	0.38	0.707

## Discussion

Since the original identification of the CAYP1A1 polymorphisms, a number of studies have investigated the genetic effect of the polymorphisms on susceptibility to human complex diseases, such as various cancers [33], [34], polycystic ovary syndrome [35], chronic kidney disease [36], coronary artery disease [37], and systemic lupus erythematosus [38]. Accordingly, the association between polymorphisms of CPY1A1 gene and susceptibility to male infertility was also reported in different populations. For example, Vani et al. [28] and Chen et al. [29] found that individuals with CYP1A1 mutations had significantly increased risk for the development of IMI in Indian and Chinese populations, whereas Salehi et al [31] and Yarosh et al. [32] revealed that CYP1A1 variants had no effect on the genetic susceptibility to the disease in Iranian and Russian populations. This inconsistency could be due to many factors, including various recruitment procedures of the study populations and differences in the genetic and environmental backgrounds. Considering the limitations from individual study, the meta-analysis of the published studies was conducted.

To the best of our knowledge, this is the first meta-analysis which comprehensively assessed the association between CYP1A1 3801T>C polymorphism and IMI risk. The principal result showed that the 3801T>C variant was associated with IMI risk in allele comparison, homozygous and recessive genetic models. In addition, the sensitivity analyses and publication bias results confirmed the robustness of these conclusions. Thus, the positive findings indicated that the CYP1A1 3801T>C polymorphism might be a potential risk factor for IMI. However, the reason for the negative findings in further subgroup analyses may be caused by the limited studies and population numbers of Caucasians and Asians included in the meta-analysis, this may have been insufficient statistical power to detect slight associations in stratified analyses.

Up to now, the underlying biological mechanism by which CYP1A1 exerts its effects on the pathogenesis of IMI is still not clearly elucidated. Recently, the important influence of estrogen in the development of male infertility has been acknowledged [32], [39]. It is well recognized that estrogens are metabolized by CYP1A1 (as an estrogen-metabolizing gene) and converted into catecholestrogens 2-hydroxyestradiol and 4-hydroxyestradiol [40]. CYP1A1 is also involved in inactivation of xenobiotic metabolism, and activation of environmental toxins. There is a complex interaction circuit between CYP1A1, estrogen receptor alpha and aryl hydrocarbone receptor with anti-estrogenic properties [41], [42]. CYP1A1 is induced by diverse exogenous and endogenous chemicals through the aryl hydrocarbone receptor [43]. Moreover, CYP1A1 expression interacts with the aryl hydrocarbone receptor and estrogen receptor alpha expression [44]. Notably, the CYP1A1 3801T>C polymorphism can alter activity and expression of the enzyme [10], [11], further regulate the expression level of aryl hydrocarbone receptor and estrogen receptor alpha, resulting in male reproduction disorders. In addition, association of CYP1A1 and estrogen polymorphisms with impaired spermatogenesis implies that both genetic and environmental factors contribute to testicular dysfunction, which can lead to sperm damage, deformity, and eventually male infertility [25]. This may be the underlying mechanism by which the CYP1A1 3801C allele resulted in male infertility.

Our study had several important strengths. Only studies in HWE among controls were included, which guaranteed quality control. Moreover, all relevant studies published in both English and Chinese were recruited for the meta-analysis, which would reduce language biases. In addition, the methodological issues for meta-analysis, such as subgroup analysis, publication bias, and stability of results were all well investigated.

In spite of several important strengths of the present meta-analysis, potential limitations should be considered. First, the results should be interpreted with caution because of obvious heterogeneity in most comparisons, although some stratified analyses by quality score and sample size did partly identify the source of heterogeneity for the 3801T>C polymorphism under homozygous model and dominant model. However, it is widely accepted that potential heterogeneity from genetic backgrounds may interfere with the conclusions of meta-analyses [45]. In our meta-analysis, there is a differential population impact depending on the 3801C allele frequency in the background population. In particular, it is evident that 3801C allele frequency is highest in Chinese [27] and Iranian [31], with much lower frequency being recorded in Russian [32] and Indian [28]. Clearly, this discrepancy may have interfered with our results. Second, lacking information of other confounding factors (e.g. age, family history, environmental factors and lifestyles) in the eligible studies limited our further stratified analysis to explore the possible sources of heterogeneity. Third, other ethnic decent studies were absent in our study, for example, Africans and African-Americans, which may have biased our results. Thus, we are not sure whether there is a significant association between the CYP1A1 3801T>C polymorphism and increased male infertility risk in the whole population. Fourth, for each selected study, our results were based primarily on unadjusted effect estimates and confidence intervals, while a more precise analysis should be conducted if all individual raw data were available. Fifth, diagnostic criteria of semen specimens in cases and definition of control subjects (including matching variables, such as age, smoking status, and alcohol intake) were differed in the meta-analyzed studies, which is of concern as inconsistent phenotype definitions may weaken the validity of meta-analysis. Therefore, more precisely defined case–control phenotypes in future studies may not only improve efficiency and validity in individual studies, but also for the overarching meta-analysis, and should be a consideration in study design. Sixth, the corresponding authors of these studies [8], [25], [26] without adequate reporting of genotype frequency were contacted for additional information, but unfortunately did not respond by the time of analysis and writing, and the papers were thus excluded, which also limits the ability to synthesize all available evidence. Noticeably, all the three previous studies were reported that the increased frequencies of CYP1A1 3801C allele contributed to risk of IMI. In a future study, we would conduct a new meta-analysis that includes the studies to further strengthen the robustness of the current meta-analysis findings. Finally, all included studies were case-control design, which precludes further comments on cause-effect relationship. The results of long-term prospective, designed for the investigation of gene-gene and gene-environment interactions, in different ethnic populations might produce more conclusive claims about the association between CYP1A1 and IMI risk.

## Conclusion

In summary, the present meta-analysis provides evidence of a significant association between CYP1A1 3801T>C polymorphism and IMI risk. However, given the limitation of the eligible studies, larger well-designed case-control or cohort studies are necessary to draw comprehensive and true conclusions.

## Supporting Information

Figure S1 **Forest plots for the association between the 3801T>C polymorphism of CYP1A1 and risk of idiopathic male infertility.** The contribution of each study to the meta-analysis (its weight) is represented by the area of a box, the center of which represents the size of the OR estimated from that study. The 95% CI for the OR (extending lines) from each study is also shown. The overall OR is shown in the middle of a diamond, the left and right extremes of which represent the corresponding CI. A: allele model, B: heterozygous model, C: dominant model, D: recessive model.
(TIF)

Figure S2 **Funnel plots for the 3801T>C polymorphism of CYP1A1 in idiopathic male infertility.** The vertical axis represents log (OR), and the horizontal axis refers to the standard error of log (OR). The horizontal line indicates the pooled OR, and the sloping lines indicate the expected 95% CI for a given standard error. The area of each circle represents the contribution of the study to the pooled OR. a: homozygous model, b: heterozygous model, c: dominant model, d: recessive model.
(TIF)

Table S1 **Details of reasons for exclusion of studies from meta-analysis.**
(DOC)

Table S2 **Main characteristics of studies included in the meta-analysis.**
(DOC)

Table S3 **Methodological quality of included case–control studies based on the Newcastle–Ottawa Scale.**
(DOC)

Checklist S1 **Prisma checklist.**
(DOC)
