# Supplementary figures and images for: Association between 3801T>C Polymorphism of CYP1A1 and Idiopathic Male Infertility Risk: A Systematic Review and Meta-Analysis
Source: PLoS One. 2014 Jan 21;9(1):e86649. doi: 10.1371/journal.pone.0086649 (PMC3897750; doi:10.1371/journal.pone.0086649)

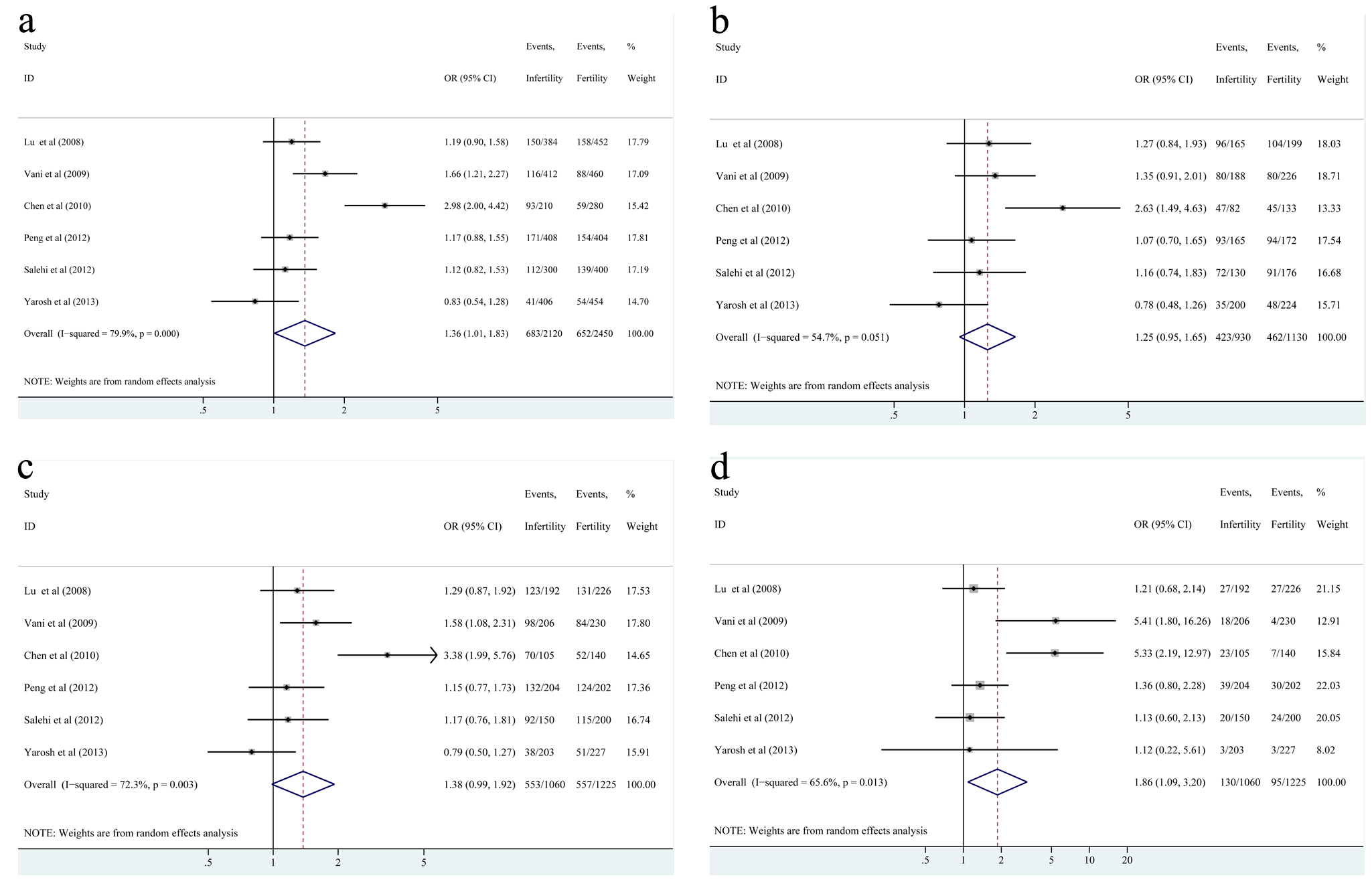

Supplement: Figure S1 — Forest plots for the association between the 3801T>C polymorphism of CYP1A1 and risk of idiopathic male infertility. The contribution of each study to the meta-analysis (its weight) is represented by the area of a box, the center of which represents the size of the OR estimated from that study. The 95% CI for the OR (extending lines) from each study is also shown. The overall OR is shown in the middle of a diamond, the left and right extremes of which represent the corresponding CI. A: allele model, B: heterozygous model, C: dominant model, D: recessive model. (TIF) [file pone.0086649.s001.tif]

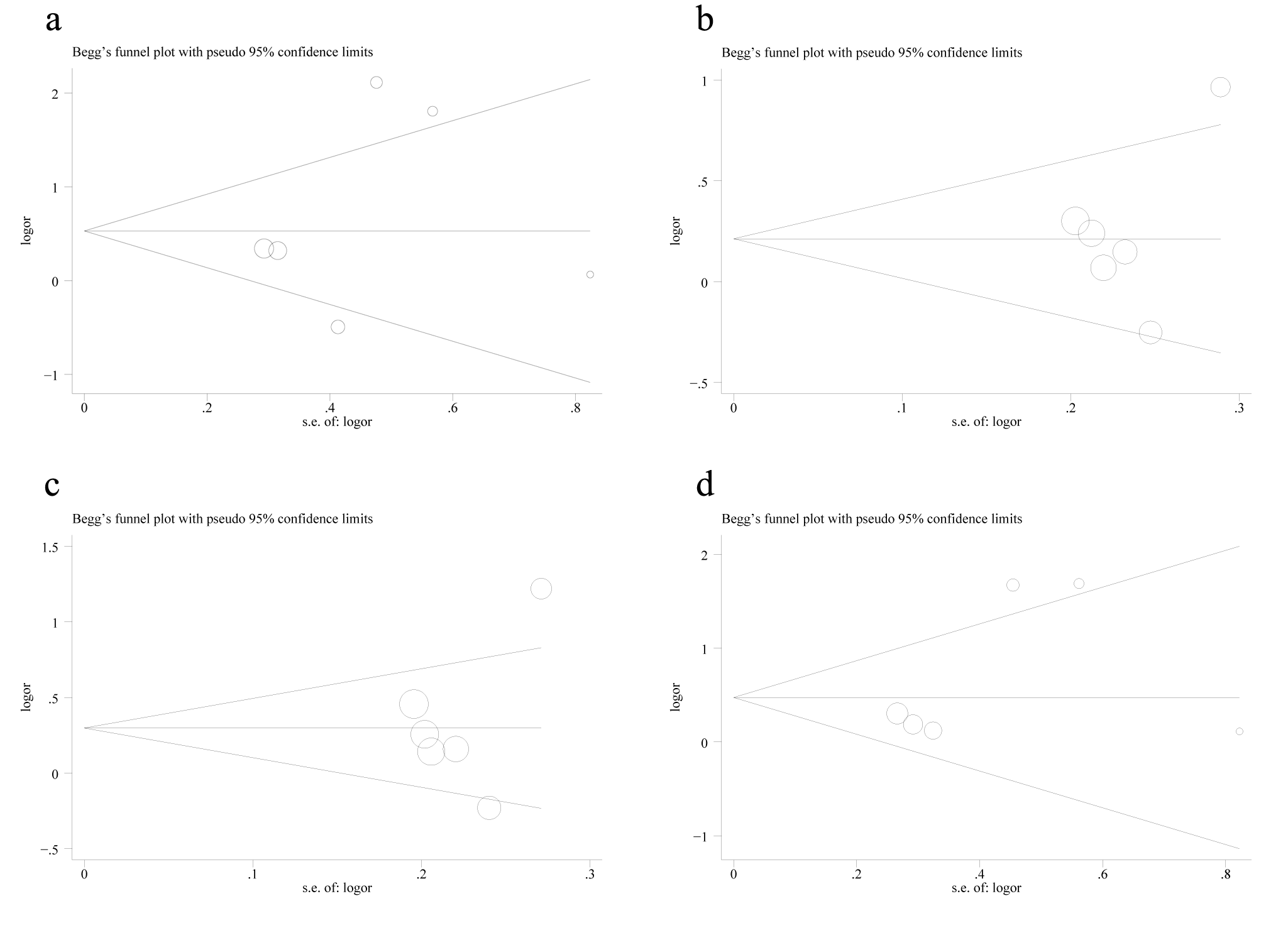

Supplement: Figure S2 — Funnel plots for the 3801T>C polymorphism of CYP1A1 in idiopathic male infertility. The vertical axis represents log (OR), and the horizontal axis refers to the standard error of log (OR). The horizontal line indicates the pooled OR, and the sloping lines indicate the expected 95% CI for a given standard error. The area of each circle represents the contribution of the study to the pooled OR. a: homozygous model, b: heterozygous model, c: dominant model, d: recessive model. (TIF) [file pone.0086649.s002.tif]
