# Supplementary material for: Association between 3801T>C Polymorphism of CYP1A1 and Idiopathic Male Infertility Risk: A Systematic Review and Meta-Analysis
Source: PLoS One. 2014 Jan 21;9(1):e86649. doi: 10.1371/journal.pone.0086649 (PMC3897750; doi:10.1371/journal.pone.0086649)
Supplement: Table S1 — Details of reasons for exclusion of studies from meta-analysis. (DOC) [file pone.0086649.s003.doc]

Table S1 Details of reasons for exclusion of studies from meta-analysis

| References | Reason for exclusion |
| --- | --- |
| 1 – 7 | Experimental articles or functional research |
| 8-18 | Other diseases |
| 19 -23 | Gene-environment interaction |
| 24-27 | Other animals |
| 28 -30 | Other variants |
| 31 | Letter |
| 32-34 | Dissertations |

**References**

1. Abass K, Reponen P, Turpeinen M, Jalonen J, Pelkonen O (2007) Characterization of diuron N-demethylation by mammalian hepatic microsomes and cDNA-expressed human cytochrome P450 enzymes. Drug Metabolism and Disposition 35: 1634-1641.

2. Arslan S, Ozgun O, Sen A (2010) Differential effects of cyclamen extract on P450 gene expression. Drug Metabolism Reviews 42: 177-178.

3. Goetz AK, Dix DJ (2009) Mode of action for reproductive and hepatic toxicity inferred from a genomic study of triazole antifungals. Toxicol Sci 110: 449-462.

4. Kohen P, Henriquez S, Rojas C, Gerk PM, Palomino WA, et al. (2013) 2-Methoxyestradiol in the human corpus luteum throughout the luteal phase and its influence on lutein cell steroidogenesis and angiogenic activity. Fertility and Sterility 100: 1397-1404.e1391.

5. Nestler D, Risch M, Fischer B, Pocar P (2007) Regulation of aryl hydrocarbon receptor activity in porcine cumulus - Oocyte complexes in physiological and toxicological conditions: The role of follicular fluid. Reproduction 133: 887-897.

6. Ozgun O, Arslan S, Sen A (2011) Differential effects of cyclamen extract on p450 gene expression in human hepatocarcinoma hepg2 cells. FEBS Journal 278: 225.

7. Yamada H, Sata F, Saijo Y, Kishi R, Minakami H (2005) Genetic factors in fetal growth restriction and miscarriage. Seminars in Thrombosis and Hemostasis 31: 334-345.

8. Akgu S, Derman O, Aktaxs D, Alikaxsifoglu M (2010) CYP1A1 gene polymorphism in Turkish adolescents with polycystic ovary syndrome. Journal of Pediatric and Adolescent Gynecology 23: e105-e106.

9. Babu KA, Rao KL, Kanakavalli MK, Suryaharayana VV, Deenadayal M, et al. (2004) CYP1A1, GSTM1 and GSTT1 genetic polymorphism is associated with susceptibility to polycystic ovaries in South Indian women. Reproductive BioMedicine Online 9: 194-200.

10. Babu KA, Reddy NGP, Deendayal M, Kennedy S, Shivaji S (2005) GSTM1, GSTT1 and CYP1A1 detoxification gene polymorphisms and their relationship with advanced stages of endometriosis in South Indian women. Pharmacogenetics and Genomics 15: 167-172.

11. Esinler I, Aktas D, Otegen U, Alikasifoglu M, Yarali H, et al. (2008) CYP1A1 gene polymorphism and polycystic ovary syndrome. Reproductive BioMedicine Online 16: 356-360.

12. Fazleabas A, Joshi N, Gadisetti M, Chen J, Ho SM, et al. (2012) Epigenetic modifications in the eutopic endometrium (EUE) of women with endometriosis and infertilty. Reproductive Sciences 19: 139A.

13. Gada RP, Tabbaa ZM, Lomberk GA, Buttar NS, Urrutia RA, et al. (2011) Uterine transcription factor KLF16 regulates endometrial physiology and metabolism via cytochrome p450 enzyme inhibition. Fertility and Sterility 96: S145.

14. Huber JC, Schneeberger C, Tempfer CB (2002) Genetic modeling of estrogen metabolism as a risk factor of hormone-dependent disorders. Maturitas 41 Suppl 1: S55-64.

15. Kandukuri LR, Suryanarayana V, Deenadayal M, Singh L (2009) Evaluation of critical genetic variation in idiopathic recurrent miscarriages among South Indian women- A genomic and proteomic approach. Maturitas 63: S132.

16. Rozati R, Giragalla SB, Bakshi H, Doddamaneni S, Khaja N, et al. (2008) The CYP1A1 and GSTM1 genetic polymorphisms and susceptibility to endometriosis in women from South India. International Journal of Fertility and Sterility 2: 105-112.

17. Suryanarayana V, Deenadayal M, Singh L (2004) Association of CYP1A1 gene polymorphism with recurrent pregnancy loss in the South Indian population. Human Reproduction 19: 2648-2652.

18. Tsuchiya M, Tsukino H, Iwasaki M, Sasaki H, Tanaka T, et al. (2007) Interaction between cytochrome P450 gene polymorphisms and serum organochlorine TEQ levels in the risk of endometriosis. Molecular Human Reproduction 13: 399-404.

19. Depalo R, Lippolis C, Vacca M, Nardelli C, Selvaggi L, et al. (2012) Influence of environmental chemical contamination on Aryl hydrocarbon Receptor in Granulosa Cells of women undergoing IVF Preliminary data. Human Reproduction 27.

20. Esakky P, Hansen DA, Drury AM, Moley KH (2012) Cigarette smoke condensate induces aryl hydrocarbon receptor-dependent changes in gene expression in spermatocytes. Reprod Toxicol 34: 665-676.

21. Hombach-Klonisch S, Pocar P, Kauffold J, Klonisch T (2006) Dioxin exerts anti-estrogenic actions in a novel dioxin-responsive telomerase-immortalized epithelial cell line of the porcine oviduct (TERT-OPEC). Toxicological Sciences 90: 519-528.

22. Omurtag K, Esakkay P, Hansen D, Moley K (2012) AHR signaling and induction of free radical scavengers in spermatocytes exposed to cigarette smoke. Reproductive Sciences 19: 124A.

23. Qin Y, Chen M, Wu W, Xu B, Tang R, et al. (2013) Interactions between urinary 4-tert-octylphenol levels and metabolism enzyme gene variants on idiopathic male infertility. PLoS One 8: e59398.

24. Diawaraa MM, Chavez KJ, Simpleman D, Williams DE, Franklin MR, et al. (2001) The psoralens adversely affect reproductive function in male wistar rats. Reprod Toxicol 15: 137-144.

25. Harvey CN, Uzumcu M (2010) Methoxychlor and methoxychlor metabolite HPTE regulate estrogen receptors expression in rat ovarian granulosa cells in vitro. Biology of Reproduction 83.

26. Murai T, Mori Y, Tatematsu K, Koide A, Hagiwara A, et al. (2005) Differences in susceptibility to N-butyl-N-(4-hydroxybutyl)nitrosamine-induced urinary bladder carcinogenesis between SD/gShi rats with spontaneous hypospermatogenesis and SD/cShi rats with spontaneous hydronephrosis. Cancer Science 96: 637-644.

27. Petroff BK, Valdez KE, Brown SB, Piasecka J, Albertini DF (2011) The aryl hydrocarbon receptor agonist 2,3,7,8-tetrachloro-dibenzo-p-dioxin (TCDD) alters early embryonic development in a rat IVF exposure model. Reproductive Toxicology 32: 286-292.

28. Mijal RS, Wirth JJ, Messaros B, Diamond MP, Friderici K, et al. (2011) Effect of smoking on human sperm parameters is modified by glutathione-S-transferase (GST) T1 genotype. American Journal of Epidemiology 173: S295.

29. Wirth JJ, Mijal RR, Friderici K, Daly DD, Paneth N, et al. (2011) Modification of the relationship between smoking and human sperm parameters by a polymorphism in glutathione-S-transferase T1. Fertility and Sterility 96: S6.

30. Tang KF, Xing JP, Sun F. Glutathione S-transferase polymorphisms and male infertility [J]. Zhonghua Nan Ke Xue Za Zhi,2013, 03:266-269.

31. Niederberger C (2012) Re: Analysis of GSTM1, GSTT1, and CYP1A1 in Idiopathic Male Infertility. J Urol 187: 1376.

32. Lu NX. Studies on the association of Polymorphisms in CYP1A1 gene and P53 gene with male infertility [D]. Nanjing Medical University, 2007.

33. Liang J. Studies on the association of XPC gene polymorphisms and spermatogenic failure [D]. Nanjing Medical University, 2008.

34. Liu B.Study on relationship between genetic polymorphisms of CYP1A1 and GSTM1 gene and the suseeptibility to teratospermia with smoke [D].Zhongnan University, 2010.
