# Supplementary material for: Association between 3801T>C Polymorphism of CYP1A1 and Idiopathic Male Infertility Risk: A Systematic Review and Meta-Analysis
Source: PLoS One. 2014 Jan 21;9(1):e86649. doi: 10.1371/journal.pone.0086649 (PMC3897750; doi:10.1371/journal.pone.0086649)
Supplement: Table S2 — Main characteristics of studies included in the meta-analysis. (DOC) [file pone.0086649.s004.doc]

Table S2 Main characteristics of studies included in the meta-analysis

| Study | Year | Country | Ethnicity | sample size (case/control) | Criteria for inclusion/exclusion in cases | Criteria for inclusion/exclusion in controls | Matching variable in cases and controls | Main diagnostic criteria |
| --- | --- | --- | --- | --- | --- | --- | --- | --- |
| Lu et al [24] | 2008 | China | Asian | 192/226 | Infertile males with azoospermia or severe oligozoospermia, a normal 46, XY karyotype, normal sexual and ejaculatory functions and without Y chromosome deletions, abnormal karyotypes, hypogonadotropic hypogonadism, seminal tract obstruction, or varicocele. | Control subjects had fathered at least one child without assisted reproductive technologies. | Age ^†^ | WHO guidelines |
| Vani et al [25] | 2009 | India | Caucasian | 206/230 | Infertile males with unknown causes (idiopathic). Infertility with known causes (cytogenetic, hormonal, Y-chromosomal deletions and female factors) was excluded. | Fertile subjects who fathered at least one child without assisted reproductive technologies. | Age, socioeconomy | WHO guidelines and criteria of Kruger |
| Chen et al [26] | 2010 | China | Asian | 105/140 | Infertility for 2 to 8 years of normal sexual and without Abnormal karyotypes, antisperm autoantibodies, hormonal, and seminal tract obstruction. | Fertile healthy subjects with normal semen parameters had fathered at least one child. | Age | NR |
| Peng et al [27] | 2012 | China | Asian | 204/202 | Male infertility with normal sexual and ejaculatory functions and without Y chromosome deletions, abnormal karyotypes, hypogonadotropic hypogonadism, seminal tract obstruction, or varicocele. | Fertile healthy subjects had fathered at least one child. | Age | NR |
| Salehi et al [28] | 2012 | Iran | Caucasian | 150/200 | An infertility history of at least 2 years with spouses. Male infertility without azoospermia, seminal infections, varicocele, systemic diseases, previous cryptorchidism or orchitis, presence of antisperm autoantibodies, hypogonadotrophic hypogonadism, abuse of androgenic (anabolic) steroids, testicular tumors, and karyotype abnormalities. | Control subjects had fathered at least one child. | Age | WHO guidelines |
| Yarosh et al [29] | 2013 | Russia | Caucasian | 203/227 | Infertility for at least 12 months of regular unprotected intercourse, with at least 2 repeated finding of semen parameter abnormalities and the negative mixed agglutination reaction test. Clinical, laboratory, and instrumental investigations by experienced andrologist, endocrinologist, geneticist, and laboratory assistants covered the exclusion of all recognizable causes of male infertility. The female infertility factor was excluded | Male volunteers (all men had fathered at least 1 child) with normal semen parameters to serve as the fertile control samples. | Smoking | WHO guidelines |

^†^ original from the author’s dissertation; NR data were not reported
