# Supplementary material for: Association between 3801T>C Polymorphism of CYP1A1 and Idiopathic Male Infertility Risk: A Systematic Review and Meta-Analysis
Source: PLoS One. 2014 Jan 21;9(1):e86649. doi: 10.1371/journal.pone.0086649 (PMC3897750; doi:10.1371/journal.pone.0086649)
Supplement: Table S3 — Methodological quality of included case–control studies based on the Newcastle–Ottawa Scale. (DOC) [file pone.0086649.s005.doc]

Table S3 Methodological quality of included case–control studies based on the Newcastle–Ottawa Scale

| Study | Year | Selection | Comparability | Exposure | Total score |
| --- | --- | --- | --- | --- | --- |
|
| Lu et al [24] | 2008 | 3 | 1 | 2 | 6 |
| Vani et al [25] | 2009 | 3 | 2 | 2 | 7 |
| Chen et al [26] | 2010 | 2 | 1 | 2 | 5 |
| Peng et al [27] | 2012 | 2 | 1 | 2 | 5 |
| Salehi et al [28] | 2012 | 3 | 1 | 2 | 6 |
| Yarosh et al [29] | 2013 | 4 | 2 | 2 | 8 |
